# Outbreak of Severe Hypoglycemia After Ingestion of a Male Enhancement Supplement — Virginia, August–November 2019

**DOI:** 10.15585/mmwr.mm6924a3

**Published:** 2020-06-19

**Authors:** Jennifer A. Ross, John W. Downs, Lindsay A. Bazydlo, Paige H. Bordwine, Catherine E. Gineste, Marissa C. Kopatic, Saumitra V. Rege, Dawn M. Saady, Okey F. Utah, Shane A. Wyatt, Brandon K. Wills, S. Rutherfoord Rose, Christopher Holstege

**Affiliations:** ^1^Department of Emergency Medicine, University of Virginia School of Medicine, Charlottesville, Virginia; ^2^Virginia Poison Center, Virginia Commonwealth University Health System, Richmond, Virginia; ^3^Department of Pathology, University of Virginia School of Medicine, Charlottesville, Virginia; ^4^Virginia Department of Health, Richmond, Virginia; ^5^Consolidated Laboratory Services, Virginia Department of General Services, Richmond, Virginia.

In August 2019, the Virginia Poison Center (VPC) and the Blue Ridge Poison Center (BRPC) were contacted concerning patients experiencing repeated episodes of marked hypoglycemia following ingestion of a male enhancement supplement tablet marketed as “V8” in convenience stores in central Virginia. Over the following 3 months, the Virginia Department of Agriculture and Consumer Services (VDACS) and the Virginia Department of Health (VDH) conducted an investigation and identified 17 patients meeting the case definition (severe hypoglycemia within 48 hours of consuming an over-the-counter male enhancement supplement in a man with no history of use of insulin or other medication used to control blood glucose). Analysis of the V8 tablets revealed that most contained glyburide, a sulfonylurea oral hypoglycemic used in the treatment of diabetes and associated with prolonged hypoglycemia following overdose (*1*). To stem this outbreak, V8 was removed from stores when found, and public service announcements were released. The public health implications of V8 use include the potential for substantial morbidity from hypoglycemic episodes and the potential for mortality if health care services are not accessed in a timely manner when hypoglycemia occurs. The presence of V8 in the market poses a serious threat to public health because of its potentially life-threatening adverse effects.

## Initial Cases

On August 13, 2019, VPC was consulted by an emergency physician at an academic medical center about a man aged 57 years who did not have diabetes and was noted by his wife to have been diaphoretic and agitated the previous day. His symptoms initially resolved after eating lunch but returned later in the day, and he became increasingly agitated. After 12 hours of confusion, he was evaluated in a hospital emergency department, where a basic metabolic panel revealed a blood glucose of 48 mg/dL (normal = 70–100 mg/dL). His mental status returned to baseline following administration of intravenous dextrose and 100 mg of octreotide, a drug that inhibits insulin release and is used as an antidote for recurrent hypoglycemia associated with sulfonylurea toxicity. The man had no known exposure to insulin or other hypoglycemic medications; however, he disclosed recently using V8, an oral male enhancement supplement purchased from a local convenience store in the metropolitan Richmond area. He had been unable to fill his usual prescription for sildenafil (used to treat erectile dysfunction) because of health insurance difficulties and reported ingesting one V8 tablet nightly during August 10–12. The treating physician and poison center suspected sulfonylurea poisoning after a literature review noted a 2009 outbreak of hypoglycemia associated with contaminated counterfeit sildenafil. A sample of the V8 product was collected for testing.

On August 22, BRPC was notified about a man aged 50 years in Lynchburg, Virginia, who did not have diabetes and who was diaphoretic, tremulous, and confused. The local emergency medical services team found his blood glucose to be 32 mg/dL, and he received oral glucose. On arrival to the emergency department, his blood glucose level was in the normal range, but hypoglycemia recurred 1 hour later. He was admitted to a hospital, where his overnight blood glucose levels dropped as low as 42 mg/dL despite intravenous infusion of dextrose-containing fluids and frequent dextrose boluses. He required hospitalization for 3 days for recurring episodes of hypoglycemia. The patient reported no use of insulin or other hypoglycemic medications; however, he did disclose recent use of the V8 supplement, purchased from a local service station in Lynchburg. Because of his prolonged hypoglycemia, the poison center hypothesized that the supplement contained a sulfonylurea. Glyburide and sildenafil were detected in the patient’s blood and urine using liquid chromatography quantitative time of flight mass spectrometry (LC-QTOF-MS), and a sample V8 tablet was collected from his personal inventory for testing.

## Outbreak Investigation and Findings

On August 14, an outbreak investigation was launched by VDACS and VDH. A confirmed case of V8-associated hypoglycemia was defined as the development of severe hypoglycemia within 48 hours of consuming an over-the-counter male enhancement supplement in a man with no history of use of insulin or other medication used to control blood glucose. During the 3 months following identification of the first two cases, 15 additional patients were hospitalized for management of hypoglycemia associated with ingestion of V8. All were men ranging in age from 33 to 73 years, and all met the confirmed case definition (Table). The mean blood glucose level for all confirmed cases at initial evaluation was 30 mg/dL, and the lowest documented level was 11 mg/dL. Three patients had two separate hospitalizations each for recurring hypoglycemia related to use of the supplement. All patients received intravenous dextrose for acute management, and seven also received octreotide. One patient received steroids and two sessions of empiric hemodialysis, although these therapies are not generally recommended for sulfonylurea poisoning. No V8-related deaths were identified. Patients reported that the V8 supplement was sold in service stations and convenience stores in clear jars without an ingredient list or warning label (Figure). The blue tablets found inside closely resembled prescription sildenafil. Patients reported that the supplement was promoted by word of mouth and was purported to enhance male sexual performance.

**TABLE:** Demographic and clinical data for confirmed cases (N = 17) of hypoglycemia associated with consumption of “V8,” an over-the-counter male enhancement supplement — Virginia, August–November 2019

Patient (visit no.)	Age (yrs)	Date of ED visit	Lowest blood glucose (mg/dL)	No. of days hospitalized	Reported duration of V8 use before ED visit	Additional treatments*	Regional poison center
A	57	08/13/19	48	1	1–3 days	octreotide	VPC
B	63	08/14/19	26	3	>1 month	octreotide	VPC
C	38	08/20/19	34	1	1–3 days	octreotide	VPC
D (1)^†^	52	08/22/19	11	6	>1 month	corticosteroids, empiric hemodialysis	BRPC
E	50	08/23/19	32	3	>1 month	None	BRPC
F	46	08/26/19	66	3	7 days	None	BRPC
G	57	08/28/19	18	3	1–3 days	None	BRPC
D (2)^†^	52	09/02/19	29	5	1–3 days	octreotide, empiric hemodialysis	BRPC
H	69	09/02/19	38	4	1–3 days	octreotide	BRPC
I	33	09/07/19	30	2	Unknown	octreotide	VPC
J (1)^†^	63	09/08/19	18	2^§^	1–3 days	None	BRPC
J (2)^†,¶^	63	09/09/19	16	2	1–3 days	None	BRPC
K	58	09/15/19	36	4	Unknown	None	VPC
L	40	09/16/19	29	1^§^	>1 month	None	BRPC
M	64	09/16/19	22	2	1–3 days	None	BRPC
N	49	10/06/19	39	3	1–3 days	None	BRPC
O (1)^†^	73	10/26/19	35	0	1–3 days	None	BRPC
O (2)^†^	73	10/27/19	22	3	1–3 days	None	BRPC
P	44	10/29/19	18	2^§^	1–3 days	None	BRPC
Q	34	11/06/19	47	2	Unknown	octreotide	VPC

**Abbreviations:** BRPC = Blue Ridge Poison Center; ED = emergency department; VPC = Virginia Poison Center.

* All patients received intravenous dextrose as needed for emergency treatment of hypoglycemia.

^†^ Patients D, J, and O each had two separate ED evaluations for medical care. Patients D and O resumed use of V8 following their first visit for medical care and suffered recurrent hypoglycemic episodes. It is unknown whether patient J resumed use of V8 after initial medical care.

^§^ Left against medical advice.

^¶^ Found with altered mental status the same day after leaving against medical advice.

**FIGURE:**
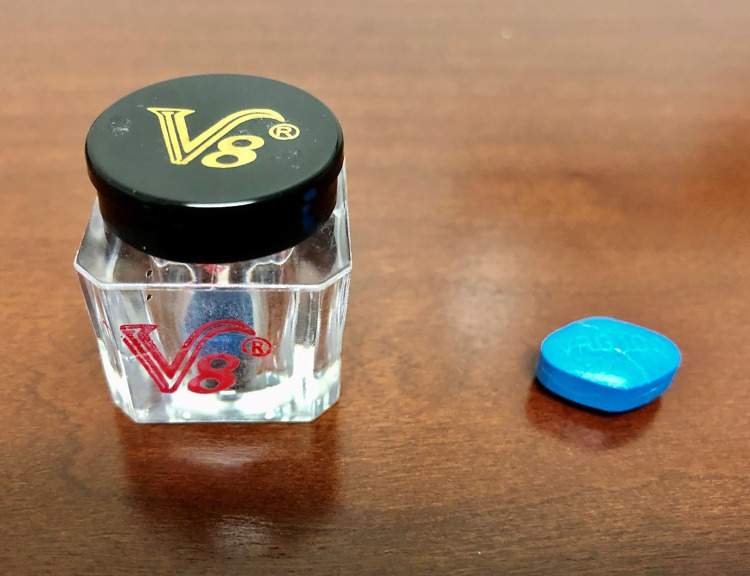
A jar of “V8,” a male enhancement supplement of blue tablets closely resembling prescription sildenafil, purchased from a convenience store — Virginia, 2019 Photo/Virgina Poison Center

Samples of V8 were obtained from the patients’ personal inventories and from several stores throughout Virginia. Tablets were independently analyzed by the Virginia Division of Consolidated Laboratory Services, the state public health laboratory using liquid chromatography with high-resolution mass spectrometry, and the University of Virginia Department of Pathology’s Laboratories using LC-QTOF-MS. The laboratories confirmed that all tablet samples contained sildenafil in amounts ranging from 55 to 156 mg per tablet, and that most tablets contained 90 to 100 mg of glyburide, a dose 5 to 10 times higher than that used in the treatment of diabetes. Blood from three patients was analyzed for the presence of glyburide, and all three tested positive. Glyburide and sildenafil were detected in the urine of a fourth patient.

## Public Health Response

VPC notified the Richmond City Health Department about the first two patients on August 14. Additional assistance was requested from VDH and VDACS to investigate the retail facilities selling V8 and initiate seizure or quarantine of these potentially glyburide-contaminated products. On August 22, VDACS released an initial public service announcement to warn consumers of potentially life-threatening hypoglycemia associated with the use of V8 supplements. Follow-up announcements were released by BRPC on August 26 and September 12, urging consumers not to use the V8 supplement. On September 16, VDH posted a notification to other states on CDC’s Epidemic Information Exchange (Epi-X). A statewide press release followed on September 17. The final confirmed case reported to VDH occurred on November 6.

Reports to MedWatch, the Food and Drug Administration (FDA) Safety Information and Adverse Event Reporting Program (https://www.fda.gov/safety/medwatch-fda-safety-information-and-adverse-event-reporting-program), were filed as cases occurred. The VDACS investigation into retail facilities resulted in product seizure at 23 locations across Virginia. An FDA investigation into the origin of these products is ongoing.

## Discussion

Over-the-counter supplements have been documented to contain prescription pharmaceuticals or other potentially harmful ingredients (*2*). In 2009, an outbreak of hypoglycemia affecting 150 persons in Singapore was linked to counterfeit sildenafil contaminated with glyburide (*3*). In the outbreak described here, a nonprescription over-the-counter supplement was also documented to contain both sildenafil and glyburide. It is unclear why glyburide was used in the manufacturing of this supplement. It has been hypothesized that the glyburide was added as an available filler. However, given that the tablets contained sildenafil doses within the typical therapeutic dosing range, the inclusion of glyburide as a filler appears less likely. Alternatively, glyburide may have been used to color the tablet blue to resemble prescription sildenafil. This outbreak has major implications for public health because consumers might purchase and use these supplements without awareness of the potential for substantial morbidity (*4*).

This investigation reveals a specific instance of undeclared prescription pharmaceuticals sold at public convenience stores as a dietary supplement and highlights the importance of the role of collaboration between poison centers, treating hospitals, health departments, public health laboratories, and the state university health system for public health surveillance, detection, and response. Prompt response to the outbreak and collaboration among multiple partners likely resulted in more rapid control of the outbreak and protection of the public from greater harm. V8 supplements and other similar products pose a serious risk for injury to consumers, illustrating an emerging risk associated with tainted male enhancement products. V8 and other male enhancement supplements containing undeclared FDA-approved prescription drugs should be removed from the market expeditiously once identified, and further efforts should be made to educate consumers and clinicians about the potential dangers of over-the-counter products sold with undeclared prescription ingredients.

**Summary**

**What is already known about this topic?**

Over-the-counter products sold as dietary supplements might contain undeclared Food and Drug Administration–approved prescription pharmaceuticals that could pose a substantial health risk to consumers who believe them to be harmless.

**What is added by this report?**

An unlabeled, over-the-counter product sold in Virginia convenience stores as a male enhancement supplement contained sildenafil and glyburide, a potent hypoglycemic agent, leading to life-threatening episodes of hypoglycemia requiring prolonged hospitalization among users.

**What are the implications for public health practice?**

Numerous tainted sexual enhancement products remain on the market as over-the-counter products, placing consumers at risk for unknown health complications. Collaborative and timely surveillance and prompt intervention are required to remove products known to cause substantial morbidity.
